# Pre-Vaccination Care-Seeking in Females Reporting Severe Adverse Reactions to HPV Vaccine. A Registry Based Case-Control Study

**DOI:** 10.1371/journal.pone.0162520

**Published:** 2016-09-09

**Authors:** Kåre Mølbak, Niels Dalum Hansen, Palle Valentiner-Branth

**Affiliations:** 1 Department of Infectious Diseases Epidemiology, Statens Serum Institut, Copenhagen, Denmark; 2 Department of Computer Science, University of Copenhagen, Copenhagen, Denmark; 3 IBM Denmark, Holte, Denmark; Universidad Nacional de la Plata, ARGENTINA

## Abstract

**Background:**

Since 2013 the number of suspected adverse reactions to the quadrivalent human papillomavirus (HPV) vaccine reported to the Danish Medicines Agency (DMA) has increased. Due to the resulting public concerns about vaccine safety, the coverage of HPV vaccinations in the childhood vaccination programme has declined. The aim of the present study was to determine health care-seeking prior to the first HPV vaccination among females who suspected adverse reactions to HPV vaccine.

**Methods:**

In this registry-based case-control study, we included as cases vaccinated females with reports to the DMA of suspected severe adverse reactions. We selected controls without reports of adverse reactions from the Danish vaccination registry and matched by year of vaccination, age of vaccination, and municipality, and obtained from the Danish National Patient Registry and The National Health Insurance Service Register the history of health care usage two years prior to the first vaccine. We analysed the data by logistic regression while adjusting for the matching variables.

**Results:**

The study included 316 cases who received first HPV vaccine between 2006 and 2014. Age range of cases was 11 to 52 years, with a peak at 12 years, corresponding to the recommended age at vaccination, and another peak at 19 to 28 years, corresponding to a catch-up programme targeting young women. Compared with 163,910 controls, cases had increased care-seeking in the two years before receiving the first HPV vaccine. A multivariable model showed higher use of telephone/email consultations (OR 1.9; 95% CI 1.2–3.2), physiotherapy (OR 2.1; 95% CI 1.6–2.8) and psychologist/psychiatrist (OR 1.9; 95% CI 1.3–2.7). Cases were more likely to have a diagnosis in the ICD-10 chapters of diseases of the digestive system (OR 1.6; 95% CI 1.0–2.4), of the musculoskeletal system (OR 1.6; 95% CI 1.1–2.2), symptoms or signs not classified elsewhere (OR 1.8; 95% CI 1.3–2.5) as well as injuries (OR 1.5; 95% CI 1.2–1.9).

**Conclusion:**

Before receiving the first HPV vaccination, females who suspected adverse reactions has symptoms and a health care-seeking pattern that is different from the matched population. Pre-vaccination morbidity should be taken into account in the evaluation of vaccine safety signals.

## Introduction

Vaccination against human papilloma virus (HPV) was introduced in Denmark in 2006 and a quadrivalent HPV vaccine was included in Danish childhood vaccination programme from 2009. The primary target group is girls at the age of 12 years, but various catch-up programmes has also been offered including a free and recommended programme for young women 19–28 years from August 2012 throughout 2013 [1]. In addition, risk based self-paid HPV vaccination outside the target groups has been advocated by the Danish Society of Gynecology and Obstetrics, among others, and it is estimated that by the end of 2015 more than 0.5 million corresponding to one fifth of all Danish females has been vaccinated against HPV. Notably, 151,487 (89%) of 170,630 girls born 1996–2000 has received at least one dose of HPV vaccine.

From 2013, this programme was challenged by an increasing number of reported suspected adverse reactions [2]. Some of the reported reactions were classified as postural orthostatic tachycardia syndrome (POTS) whereas others resembled chronic fatique syndrome including fatigue, long-lasting dizziness, headache, and syncope [3,4]. The Danish safety signal was raised to the European Medicines Agency (EMA) Pharmacovigilance Risk Assessment Committee (PRAC) in September 2013. Furthermore, in July 2015, the European Commission requested PRAC to assess whether there was evidence of a causal association between HPV vaccines and POTS and/or complex regional pain syndrome (CRPS) as an association between HPV vaccine and CRPS was raised from Japan [5]. PRAC concluded that the current evidence does not support that HPV vaccines causes CRPS or POTS [6]. In December 2015 the WHO Global Advisory Committee on Vaccine Safety reported that it has not found any safety issue that would alter its recommendation of the use of HPV vaccines, and argued that despite the difficulties in diagnosing or fully characterizing CRPS and POTS, reviews of pre- and post-licensure data provide no evidence that these syndromes are associated with HPV vaccination [7].

In spite of these reports, the Danish debate about safety of HPV vaccines has not been settled, and vaccination rates are declining [8] as also seen in France and Japan [9,10]. It can be argued that many of the symptoms that have been reported by vaccinated females are non-specific and will not be captured in epidemiological studies where outcomes are based on hospital discharge diagnosis [4]. For example, headache, orthostatic intolerance, fatigue, nausea, and cognitive dysfunction were the five most commonly reported symptoms among 53 girls/women who suspected adverse events [3]. These symptoms will not be systematically recorded in registries and databases that are used for epidemiological studies. Hence, there is a need for additional studies to investigate the safety of HPV vaccines. In order to inform such studies, we aimed to elucidate the Danish signal and to obtain a better understanding of the epidemiology of the reported adverse events. The specific aim of the present study was to determine health care-seeking prior to the first HPV vaccination among females who suspected and reported adverse reactions to HPV vaccine.

## Methods

We conducted a registry-based case-control study where cases were vaccinated females with reports of suspected severe adverse reactions, and controls were vaccinated females without known reports of suspected adverse reactions. From the DMA we obtained a line-list of individuals with suspected adverse reactions. These events were reported by the patients, the parents, or their doctors, and were classified as severe adverse reactions by established criteria (mainly because the symptoms affected everyday life, e.g., not being able to attend school or work as usual, and lead to “persistent or significant disability/incapacity”) [11]. The list was obtained in August 2015, and included data on 322 individuals with a valid Danish Civil Registry Number (CPR-number) which we needed for further linkage. These individuals represented all cases classified as severe and reported with a valid CPR-number at the time of the data retrieval. After excluding one female from Greenland and five males, the dataset consisted of 316 cases.

Controls were selected from the Danish vaccination registry [12] and matched with cases in groups by year of vaccination (+/- 1 year), age at vaccination (+/- 1 year) and municipality. We included all available controls rather than aiming at a fixed number of controls per case. We obtained information on contacts to primary health care and hospital services in a two-year period prior to the first HPV vaccine. Primary health care data were obtained from The National Health Insurance Service Register. Based on reimbursement codes, contacts were categorized as (1) consultations including visits at general practitioners and specialists as well as home visits, (2) consultations by phone or e-mail, (3) laboratory analyses at a primary health care provider or by referral, (4) any type of physiotherapy or referral to chiropractor, (5) private specialist in psychiatry (including youth psychiatry) and psychologist, and (6) dental treatment.

Contacts to hospitals were obtained from the National Patients Registry. We included only main diagnoses. We parametrized diagnoses according to chapters in ICD-10 except for Z00-Z99 which was a commonly used category that was separated according to frequency of contacts into Z01.6 (radiological examination, not elsewhere classified), Z03-9 (observation for suspected disease or condition, unspecified), Z30-Z39 (persons encountering health services related to reproduction), and other contacts in this chapter.

Both for primary health care contacts and hospital diagnosis we counted one activity per group rather than calculating the number of contacts or diagnoses, i.e., if a study subject had at least one visit at the general practitioner’s office it would count as an exposure in that category and any follow-up visits were not included.

We analysed the data by logistic regression while adjusting for the matching variables; this procedure was applied as cases and controls were matched on a group basis. Due to low numbers, municipality was aggregated to provinces (nomenclature of territorial units for statistics (NUTS) code 2), year of vaccination was categorized as 2006–2009, 2010, 2011, 2012, 2013, 2014–15, and age was fitted as years except 10–12, 30–39, and 40+ years that were treated as groups due to low numbers. All these parameters were parametrized by dummy variables. For the final multivariable model, we considered variables that were associated with being a case (p-value < 10%). These variables were subsequently eliminated from the logistic regression model if they were not significant at the 5% level. Excluded variables were one-by-one re-entered in the final model and once more eliminated based on significance testing. Finally, the significant exposures from the multivariable model were examined for statistical interaction with age categorised as 10–12 years (the youngest target age for the HPV vaccination programme), 13–18 years (old girls), 19–28 (young women in the catch-up programme), and 29+ (older women). The study was notified to the Danish data protection agency under the record number 2008-54-0474.

## Results

**Fig 1:**
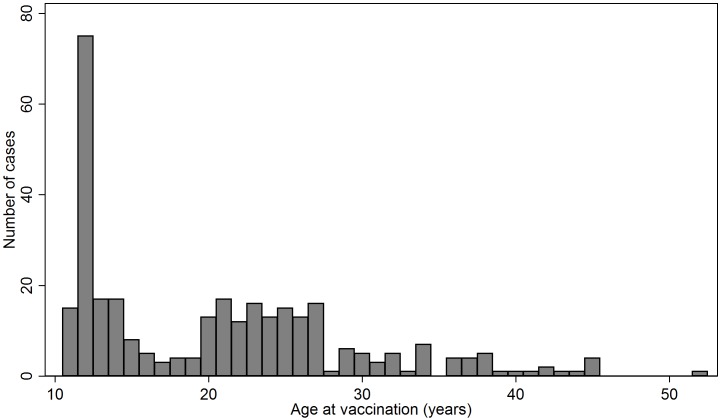
Age at first HPV vaccination for 316 females who had suspected adverse events reported to the Danish Medicines Agency and served as cases for the registry based case-control study on care-seeking prior to the first HPV vaccination.

The 316 cases had a median age of 20 years (range 11–52 years) and the 163,910 controls a median age 19 years (range 10–52 years). Fig 1 shows a bimodal age distribution of the cases with one peak around 12 years of age when childhood vaccinations are offered and another 19–28 years of age corresponding to the catch-up programme. The cases received the first vaccine between 2006 and 2014; in total 168 (53.2%) received it in 2012–2013 and 140 of these were > 18 years and therefore likely to be part of the catch-up programme offered in those two years.

Table 1 shows contacts at the primary health care level in the two years prior to vaccination. Cases had increased odds of consultations by phone or e-mail and had more laboratory analyses requested than controls. Furthermore, cases were more often referred to physiotherapy/chiropractor and psychiatry/psychologist.

**Table 1:** Primary health care contacts (assessed by reimbursement codes) two year before the first HPV vaccination in 316 females who reported suspected adverse events to the vaccine and 163,910 matched controls. Denmark, 2006 to 2015. Odds ratio is adjusted for age at vaccination, year of vaccination, and province (NUTS 2 area code).

Type of contact before first vaccination	Cases		Controls		Odds ratio	95% CI
	Number	Percent	Number	Percent		
Consultation at the office	310	98.1	157853	96.3	1.74	0.77–3.92
Consultation by phone or e-mail	299	94.6	142862	87.2	2.38	1.44–3.92
Laboratory analysis request	249	78.8	110092	67.2	1.73	1.29–2.32
Physiotherapy or related	96	30.4	20459	12.5	2.56	1.99–3.31
Psychologist, psy-chiatrist etcetera	39	12.3	9751	5.9	2.18	1.54–3.11
Dentist	134	42.4	62590	38.2	0.94	0.65–1.35

Table 2 shows that in the two years prior to vaccination there was an increased odds of diagnosis in several chapters of the ICD-10 system including the respiratory system, the digestive system, the musculoskeletal system, injuries as well as factors influencing health status and contact with the health services and symptoms, signs and abnormal findings, not elsewhere classified.

**Table 2:** Hospital contacts two year before the first HPV vaccination in 316 females who reported suspected adverse events to the vaccine and 163,910 matched controls. Denmark, 2006 to 2015. Odds ratio is adjusted for age at vaccination, year of vaccination and province (NUTS 2 area code).

Type of contact before first vaccination (ICD10 code and interpretation)	Cases		Controls		Odds ratio	95% CI
	Number	Percent	Number	Percent		
Z01.6, radiological examination, not elsewhere classified	68	21.5	18359	11.2	1.91	1.45–2.53
Z03.9, observation for suspected disease or condition, unsp.	26	8.2	5660	3.5	2.18	1.45-3-27
Z30-Z39, persons encountering health services related to reproduction	37	11.7	9413	5.7	1.35	0.92–1.98
Z00-Z99, factors influencing health status and contact with health services, *not included above*	80	25.3	21835	13.3	1.82	1.40–2.36
A00-B99, certain infections and parasitic diseases	5	1.6	2400	1.5	1.07	0.44–2.62
C00-D89, neoplasms, disease of the blood and blood forming organs and certain disorders involving immune mechanisms	6	1.9	2319	1.4	0.93	0.41–2.12
E00-E90, endocrine, nutritional and metabolic diseases	9	2.9	3368	2.1	1.18	0.60–2.30
F00-F99, mental and behavioural disorders	15	4.8	6450	3.9	1.20	0.71–2.03
G00-G99, the nervous system	9	2.9	2561	1.6	1.55	0.79–3.03
H00-H59, the eye and adnexa	7	2.2	1806	1.1	1.79	0.84–3.81
H60-H95, the ear and mastoid process	3	1.0	1483	0.9	1.01	0.32–3.19
I00-I99, the circulatory system	3	1.0	1094	0.7	0.92	0.29–2.92
J00-J99, the respiratory system	14	4.4	3958	2.4	1.81	1.05–3.10
K00-K93, the digestive system	23	7.3	5420	3.3	2.08	1.35–3.20
L00-L99, the skin and subcutaneous tissue	6	1.9	2555	1.6	1.14	0.50–2.57
M00-M99, the musculoskeletal system and connective tissue	38	12.0	8516	5.2	2.28	1.62–3.23
N00-N99, the genitourinary system	15	4.8	5875	3.6	0.93	0.55–1.59
O00-O00, pregnancy, childbirth and the puerperium	41	13.0	11037	6.7	1.37	0.95–1.98
P00-P96, conditions originating in the perinatal period	0	-	53	< 0.1	-	
Q00-Q99, congenital malformations, deformations and chromosomal abnormalities	5	1.6	2310	1.4	1.10	0.45–2.66
R00-R99, symptoms, signs and abnormal clinical and laboratory findings, not elsewhere classified	44	13.9	9709	5.9	2.33	1.68–3.22
S00-T14, injuries	102	32.3	35693	21.8	1.81	1.42–2.30
T14-T98, other injuries, poisoning and certain other consequences of external causes not included in S00-T14	11	3.5	3952	2.4	1.36	0.74–2.49
X00-X90, various external causes of accidental injury	1	0.3	93	0.1	6.77	0.93–49.45

The final multivariable model, Table 3, found independent effects of use of telephone/email consultations (Odds Ratio (OR) 1.9), physiotherapy/chiropractor (OR 2.1), and psychiatrist/psychologist (OR 1.9). Further, cases were more likely to have a diagnosis in the chapters of diseases of the digestive system (OR 1.6), of the musculoskeletal system (OR 1.6), symptoms or signs not classified elsewhere (OR 1.8), as well as injuries (OR 1.5). In total 203 (64.2%) of cases had one or more occurrences of the hospital diagnoses in Table 3 or had received reimbursement for physiotherapy, chiropractor, psychiatry or psychologist, compared with 66,222 (40.4%) of controls.

**Table 3:** Final multivariable model showing health care-seeking in the two years prior to vaccination in 316 Danish females who reported suspected adverse events to HPV vaccination compared with 163,910 matched controls. Care-seeking was defined as contact to primary health care (from registry of reimbursements) or hospital (from the Danish National Patient Registry) in a two-year period before the first HPV vaccine. All estimates were adjusted for age at vaccination, year of vaccination and province (NUTS 2 area code).

Type of contact before first HPV vaccination	Multivariable odds ratio for care-seeking	95% CI
Consultation at primary health care provider by phone or e-mail	1.91	1.15–3.16
Reimbursement of physiotherapy, chiropractor or related treatment	2.13	1.64–2.76
Reimbursement of psychologist, psychiatrist or related treatment	1.87	1.31–2.66
Hospital contact, ICD-10 code K00-K93, the digestive system	1.57	1.01–2.45
Hospital contact, ICD-10 code M00-M99, the musculoskeletal system and connective tissue	1.56	1.09–2.23
Hospital contact, ICD-10 code R00-R99, symptoms, signs and abnormal clinical and laboratory findings, not elsewhere classified	1.77	1.27–2.48
Hospital contact, ICD-10 code S00-T14, injuries	1.51	1.18–1.93

Table 4 shows the age-stratified results. Overall, there was no effect modification by age as evaluated by a formal test for interaction (p-values ranging from 0.10 to 0.78, Table 4). Thus, with the current sample size, findings were consistent across age groups. However, there was a tendency of a stronger association between exposure and outcome in the oldest age-category. In most exposure-categories, care-seeking tended to increase by increasing age with the notable exception of injuries which were more common in girls than in young women. Forty two percent of the cases 11–12 years of age had a hospital contact due to injuries compared with 27% cases, Table 4.

**Table 4:** Age specific health care-seeking in the two years prior to vaccination in 316 Danish females who reported suspected adverse events to HPV vaccination compared with 163,910 matched controls. Care-seeking was defined as contact to primary health care (from registry of reimbursements) or hospital (from the Danish National Patient Registry) in a two-year period before the first HPV vaccine. The analyses were restricted to significant variables from the final multivariable model, see Table 3.

		Cases		Controls				
Type of contact before first vaccination	Age at first HPV vaccine	Number	Percent	Number	Percent	Odds ratio*	95% CI	p-value for interaction with age**
Consultation at primary health care provider by phone or e-mail	10–12 years	78	86.7	39739	77.0	2.11	1.25–3.59	0.567
	13–18 years	51	94.4	23176	79.4	1.23	0.81–1.87	
	19–28 years	118	98.3	77003	96.1	1.92	1.28–2.90	
	29 years +	52	100.0	2944	99.2	23.54	15.21–36.46	
Reimbursement of physiotherapy, chiropractor or related treatment	10–12 years	12	13.3	3032	5.9	1.91	1.05–3.44	0.104
	13–18 years	14	25.9	2700	9.2	1.62	0.73–3.61	
	19–28 years	49	40.8	13809	17.2	1.58	0.82–3.07	
	29 years +	21	40.4	918	30.9	9.95	4.73–20.93	
Reimbursement of psychologist, psychiatrist or related treatment	10–12 years	3	3.3	520	1.0	2.54	0.81–7.97	0.486
	13–18 years	2	3.7	494	1.7	0.88	0.15–5.36	
	19–28 years	27	22.5	8409	10.5	0.87	0.26–2.93	
	29 years +	7	13.5	328	11.1	5.80	1.46–22.99	
ICD 10 code K00-K93, the digestive system	10–12 years	2	2.2	909	1.8	0.93	0.23–3.76	0.782
	13–18 years	2	3.7	652	2.2	1.71	0.24–12.27	
	19–28 years	14	11.7	3726	4.7	2.48	0.56–11.06	
	29 years +	5	9.6	133	4.5	23.32	4.41–123.31	
ICD 10 code M00-M99, the musculoskeletal system and connective tissue	10–12 years	4	4.4	1575	3.1	1.13	0.42–3.05	0.629
	13–18 years	4	7.4	1306	4.5	1.51	0.37–6.12	
	19–28 years	23	19.2	5409	6.8	2.48	0.85–7.30	
	29 years +	7	13.5	226	7.6	17.97	5.12–63.06	
ICD 10 code R00-R99, symptoms, signs and abnormal clinical and laboratory findings, not elsewhere classified	10–12 years	9	10.0	2052	4.0	1.95	1.00–3.81	0.465
	13–18 years	7	13.0	1220	4.2	1.62	0.59–4.42	
	19–28 years	21	17.5	6210	7.8	1.07	0.48–2.39	
	29 years +	7	13.5	227	7.7	9.77	3.54–26.99	
ICD 10 code S00-T14, injuries	10–12 years	38	42.2	13945	27.0	1.25	0.87–1.79	0.681
	13–18 years	24	44.4	7352	25.2	1.53	0.89–2.64	
	19–28 years	30	25.0	14028	17.5	1.14	0.68–1.91	
	29 years +	10	19.2	368	15.6	3.95	7.42–32.59	

*Note*: Cases/controls included: 10–12 years: 90/51,583; 13–18 years: 54/29,205; 19–28 years: 120/80,154; 29 years +: 52/2,968.

* Odds ratio from a logistic regression model adjusting for year of vaccination and province.

** Log-likelihood ratio test comparing main effects model with a model including main effects and an interaction term between age group and care-seeking

In the national patient registry, cases had in total 1030 discharge diagnoses (mean 3.3 per case) compared with 267,912 diagnoses among the controls (mean 1.6 per case). There were 34 diagnoses given to the 23 cases with a diagnosis of the digestive system, including haemorrhage of anus and rectum (4 occurrences) as well as functional dyspepsia and maxillary hypoplasia (3 occurrences each), gastro-oesophageal reflux disease, irritable bowel syndrome, unspecified functional intestinal disorder, unspecified anal fissure, and calculus of gallbladder without cholecystitis (all 2 occurrences). There were 73 diagnoses given to the 38 cases with a diagnosis of the musculoskeletal system included epicondylitis lateralis (7 occurrences), chondromalacia patellae, pains in limb, other chronic osteomyelitis (4 occurrences each), unspecified shoulder lesion, muscle strain, dislocation and subluxation of joint, juvenile rheumatoid arthritis, and other psoriatic arthropathies (all 3 occurrences each). There were 99 diagnoses given to the 44 cases with a code from the R-chapter, including 47 diagnoses of abdominal and pelvic pain, complex chronic non-malignant pain (7 occurrences), syncope and collapse (6 occurrences) and other and unspecified symptoms and signs involving the nervous and musculoskeletal systems (7 occurrences). There were 200 diagnoses given to the 102 cases with a diagnosis from the S00 to T14 (injuries). The most commonly reported diagnosis was sprain and strain of ankle (18 occurrences), various contusions (44 occurrences), soar and strains of fingers, wrist or knee (21 occurrences), open wound of finger (7 occurrences) and fracture of finger (5 occurrences).

## Discussion

We found that girls or women with reports of suspected adverse reactions from HPV vaccination already before their first vaccination had a health care-seeking pattern different from a matched population who did not report adverse events. For example, there was increased odds of having had injuries and being in contact with physiotherapy and chiropractor. This is compatible with the hypothesis that many cases had high levels of physical activity before onset of symptoms, as suggested by Brinth el al [3]. In a case-series (without a reference group) of 53 patients with possible adverse events Brinth et al. found that 67% had a high and 33% had a moderate physical activity level before symptom onset. Five patients were competing on a national or international level in their sport [3]. Our observation of increased number of injuries, musculoskeletal complaints and referral to physiotherapy can be interpreted as an agreement with their findings.

It is, however, unlikely that high physical activity can explain all our observations. Frequent health care attendance and the increased odds of having symptoms in the R-chapter of the ICD-10 system, including abdominal pains, syncope and other symptoms from the autonomic nervous system may point towards another hypothesis namely the presence of medically unexplained physical symptoms coinciding with the time of vaccination. Medically unexplained physical symptoms and somatoform disorders are among the most common causes of contacts with health care and is particularly common among frequent health care attenders [13,14]. Because of the high prevalence, medically unexplained physical symptoms are likely to be present in a considerable proportion of vaccinated females. The temporal association may, in the mindset of the parents or the vaccinated, be linked to vaccination.

Furthermore, cases had an increased odds ratio of contact to psychologist or psychiatrist but not an increased odds of hospital diagnoses in the F-chapter of ICD-10, mental and behavioural disorders. The most common psychiatric diagnosis was emotionally unstable personality disorder including borderline disorder but this diagnosis was not more frequent in cases than in controls.

Further studies based on larger datasets are needed to examine whether increased care-seeking is related to specific disorders in order to understand whether any specific factors may be associated with the experience of suspected adverse events.

Notably, of the first 107 claims that were reported to The Danish Patient Compensation Association, only three were recognized as adverse reactions and received compensation whereas all others were dismissed. In several of the dismissed claims, the symptoms were present before the vaccination [15].

One important strength of the study was that the assessment of care-seeking was obtained by an ongoing and unbiased collection of data to national registries. The figures are therefore reliable. Also, a number of outcomes occurred at equal odds including for example physical primary health consultations, contacts related to pregnancy and childbirths, psychiatric diagnoses given at a hospital as well as appointment at the dentists. This suggests that the case- and the control-groups were comparable. Furthermore, all reported estimates were adjusted for age at vaccination, year at vaccination and place of residence.

The study is subject to some limitations. Since August 2015 when we received the data, the number of reported possible adverse events have increased furthermore, and by the end of 2015 a total of 2019 possible adverse events have been reported to the DMA of which 823 have been classified as severe. At the present, we do not know if the 316 cases included in our study are representative of all reported cases. The primary health care data contained limited clinical information that represents another limitation. The assessment of the care-seeking behaviour to primary health care was crude and data on diagnoses and medications issued in primary health care would have added further value to the study. Furthermore, the data from primary health care was limited to activities that were reimbursed as part of the National Health Insurance Service. We did not obtain information on activities such as self-paid contacts to psychologist or school psychologists as well as physiotherapy offered in organisations such as sports clubs. Hence, the proportion of females with this type of care are minimum estimates.

The study was based on data prior to the first HPV vaccine and does not exclude that some of the females who reported suspected adverse events indeed experienced reactions caused by the vaccine. However, the study shows that the case-population as a whole was a selected population; this may in particular be the situation for those above 28 years of age. In most cases, some symptoms were present before the start of HPV vaccination. As mentioned, 140 cases (44%) had received the vaccination in the catch-up programme 2012–13 and in total 52 (16%) were older than 28 years at first vaccination and thus outside of the official programme. This may indicate that more perceived adverse events can be expected to be reported when the vaccines are offered outside of the core target group, i.e., girls before onset of sexual activity. We propose that the high vaccination-uptake in particular in the catch-up programme and beyond by coincidence led to considerable number of temporal associations between vaccination and medically unexplained physical symptoms. Teenagers and young women have, as seen in the control population of the present study, a higher proportion of ICD-10 diagnosis in the K, M and R chapters of the ICD-10 system (Table 4), and therefore a catch-up programme may have a higher risk of being challenged by the perception of adverse events than a steady routine vaccination at 12-years old.

The observed excess morbidity and excess care seeking does not rule out that the vaccine in certain situations may have triggered a course that resulted in deterioration of symptoms in some individuals in a perhaps vulnerable subpopulation, and further research should address this possible explanation. The aim was not to disentangle these and other possible trajectories; our aim was to explore the care-seeking behaviour before the first vaccination and emphasise that any conclusions as regards the safety of the vaccine should be taken based on an understanding of the characteristics of the group from which adverse events were reported.

A number of post-licensure epidemiological studies have addressed the safety of HPV vaccines, including the expected number of immune mediated disorders in girls 12–15 years prior to introduction of HPV vaccination [16]. After the introduction of the HPV vaccine the risk of a number of defined outcomes was not found to be associated with HPV vaccination in several studies [17–19]. A large cohort study from France did not find an increased incidence of the autoimmune disorders studied, with the exception of Guillain-Barré syndrome for which there was a small increased risk in the HPV vaccinated girls (~1 per 100,000 vaccinated girls) [7]. Only few studies have looked at non-specific outcomes [20,21], and we hope that our study will provide inspiration for additional epidemiological studies to address the safety of HPV vaccines, with a strong suggestion that pre-vaccination morbidity should be taken into account in the evaluation of safety signals.
